# Interleukin-36γ is expressed by neutrophils and can activate microglia, but has no role in experimental autoimmune encephalomyelitis

**DOI:** 10.1186/s12974-015-0392-7

**Published:** 2015-09-17

**Authors:** Lusine Bozoyan, Aline Dumas, Alexandre Patenaude, Luc Vallières

**Affiliations:** Axis of Neuroscience, University Hospital Center of Quebec, 2705 Laurier Boulevard, room T2-50, Quebec, QC G1V 4G2 Canada; Department of Molecular Medicine, Laval University, Quebec, Canada

**Keywords:** IL-36 gamma, IL-1 F9, IL-1Rl2, IL-1Rrp2, Granulocytes, Microglial cells, Neuroinflammation, Autoimmunity, Multiple sclerosis

## Abstract

**Background:**

Experimental autoimmune encephalomyelitis (EAE) is a model of inflammatory demyelinating diseases mediated by different types of leukocytes. How these cells communicate with each other to orchestrate autoimmune attacks is not fully understood, especially in the case of neutrophils, whose importance in EAE is newly established. The present study aimed to determine the expression pattern and role of different components of the IL-36 signaling pathway (IL-36α, IL-36β, IL-36γ, IL-36R) in EAE.

**Methods:**

EAE was induced by either active immunization with myelin peptide, passive transfer of myelin-reactive T cells or injection of pertussis toxin to transgenic 2D2 mice. The molecules of interest were analyzed using a combination of techniques, including quantitative real-time PCR (qRT-PCR), flow cytometry, Western blotting, in situ hybridization, and immunohistochemistry. Microglial cultures were treated with recombinant IL-36γ and analyzed using DNA microarrays. Different mouse strains were subjected to clinical evaluation and flow cytometric analysis in order to compare their susceptibility to EAE.

**Results:**

Our observations indicate that both IL-36γ and IL-36R are strongly upregulated in nervous and hematopoietic tissues in different forms of EAE. IL-36γ is specifically expressed by neutrophils, while IL-36R is expressed by different immune cells, including microglia and other myeloid cells. In culture, microglia respond to recombinant IL-36γ by expressing molecules involved in neutrophil recruitment, such as Csf3, IL-1β, and Cxcl2. However, mice deficient in either IL-36γ or IL-36R develop similar clinical and histopathological signs of EAE compared to wild-type controls.

**Conclusion:**

This study identifies IL-36γ as a neutrophil-related cytokine that can potentially activate microglia, but that is only correlative and not contributory in EAE.

**Electronic supplementary material:**

The online version of this article (doi:10.1186/s12974-015-0392-7) contains supplementary material, which is available to authorized users.

## Background

Experimental autoimmune encephalomyelitis (EAE) is an inflammatory demyelinating disease of the central nervous system (CNS) that can be induced in animals to model immunological processes involved in human diseases such as multiple sclerosis, neuromyelitis optica, and acute disseminated encephalomyelitis. EAE is initiated by CD4^+^ T helper lymphocytes of the Th_1_ or Th_17_ subset that recognize myelin peptides through their T cell receptors [1]. These cells do not act alone, but in concert with different myeloid cells, including monocyte-derived CD11c^+^ dendritic cells [2–5], which activate them by presenting the myelin peptides together with costimulatory signals [6–12], and monocyte-derived macrophages [2, 4, 13, 14], which execute effector functions leading to demyelination [15]. In addition, recent studies have established that neutrophils importantly contribute to EAE [16–19], although their precise role is still unclear.

To coordinate their actions, immune cells must communicate with each other through chemical messengers such as interleukins, chemokines, and growth factors. Many of these molecules have been studied in the context of EAE in the hope of identifying potential therapeutic targets. For example: GM-CSF derives from T cells and promotes dendritic cell recruitment [20, 21], IL-12 and IL-23 are secreted by dendritic cells to sustain T cell activation [22], and IL-1β is released from different myeloid cells to induce inflammatory responses [23]. However, no neutrophil-specific cytokine has been identified to date.

Following a study conducted to clarify the mechanism underlying the adjuvant effect of pertussis toxin (PTX) in EAE [24], we compared the transcriptional profiles of peritoneal leukocytes isolated from mice injected or not with PTX using DNA microarrays (unpublished data). Among the most upregulated genes was IL-36γ (also called IL1F9), a newly characterized cytokine of the IL-1 family [25]. The IL-36–IL-36R signaling pathway comprises three agonists (IL-36α, IL-36β, IL-36γ), two antagonists (IL-36RA, IL-38), one specific receptor subunit (IL-36R), and one accessory receptor subunit (IL-1RAcP) that is shared with the classical interleukin-1 receptor (IL-1R). The IL-36 proteins exhibit a typical β-trefoil structure [26] whose activity is enhanced by N-terminal truncation [27].

In the CNS, the expression of IL-36γ has never been studied, but that of IL-36R has been reported under normal conditions in non-neuronal elements such as the meninges, choroid plexus, and perivascular cells [28]. In the periphery, IL-36γ expression is constitutive in epithelial cell lines and inducible in myeloid cells (monocytes, macrophages, dendritic cells) by inflammatory mediators (e.g., IL-1β, TNF, IL-17, IL-23, TLR ligands) [29–37]. IL-36R is found on keratinocytes, splenic CD4^+^ T cells, and different myeloid cells [38–40].

IL-36γ promotes not only inflammation but also dendritic cell maturation and ability to generate a Th_1_ response. Indeed, recombinant IL-36γ induces cytokines and chemokines (e.g., IL-6, IL-8, Csf2, Csf3, Cxcl1, Ccl20) when injected into epithelial tissues, resulting in neutrophil and T cell infiltration [31, 40–42]. Furthermore, IL-36γ stimulates dendritic cells to produce inflammatory mediators (e.g., IL-6, IL-1β, IL-12p40, IL-12p35, IL-23p19, Cox-2, Cxcl1, Csf2) and cell surface receptors involved in antigen presentation (e.g., MHCII, CD80, CD83, CD86) [38–40]. IL-36γ can also induce CD4^+^ T cells to adopt an inflammatory IFNγ^+^ Th_1_ profile even in the absence of antigen-presenting cells, indicating that it can directly signal through IL-36R on T cells [38]. Consistently, the IL-36–IL-36R axis was found to be involved in psoriasis [29, 32, 34, 43–50], but the possibility that it also contributes to other T cell-driven autoimmune diseases such as multiple sclerosis remains to be determined.

The goal of the present study was to test the hypothesis that myeloid cells produces IL-36γ during EAE to promote inflammatory Th_1_/Th_17_ responses towards myelin. The findings presented below demonstrate that IL-36γ is specifically and strongly expressed by neutrophils in both CNS and hematopoietic compartments during certain forms of EAE and that microglia can react to IL-36γ by releasing neutrophil-related cytokines; however, IL-36γ does not influence the clinical and histopathological signs of EAE.

## Methods

### Animals

C57BL/6 mice were obtained from The Jackson Laboratory. Colonies of IL-36γ^–/–^, IL-36R^–/–^, and 2D2 mice (C57BL/6 background) were generated from breeders provided by the Mutant Mouse Regional Resource Center, Amgen or The Jackson Laboratory, respectively. The genotypes were confirmed by PCR using the primers listed in Additional file 1: Table S1. The experiments were performed on male mice aged 8–10 weeks under specific pathogen-free conditions with the approval of the Laval University Animal Protection Committee.

### EAE induction by active immunization

Mice were subcutaneously injected into both flanks with a total of 200 μl of emulsion containing 300 μg of MOG_35-55_ peptide (Feldan) dissolved in saline and mixed with an equal volume of complete Freund’s adjuvant containing 500 μg of killed *Mycobacterium tuberculosis* H37 RA (Difco Laboratories). They were also injected intraperitoneally with 20 μg/kg of PTX (List Biological Laboratories) immediately and 2 days after immunization.

### EAE induction by adoptive transfer

Mice were intraperitoneally injected with 20 × 10^6^ encephalitogenic cells. These were isolated from abdominal lymph nodes and spleens of mice killed 8 days after active EAE induction and then cultured for 2 days in DMEM with MOG_35-55_ peptide (15 μg/ml), murine IL-12 (5 ng/ml, R&D Systems), murine IL-23 (20 ng/ml, R&D Systems), heat-inactivated HyClone bovine growth serum (10 %, Thermo Scientific), modified Eagle’s medium non-essential amino acids (1 %, Wisent), penicillin (100 U/ml), streptomycin (100 μg/ml), and amphotericin B (250 ng/ml).

### EAE induction in 2D2 mice

2D2 mice received two intraperitoneal injections of PTX (20 μg/kg) at a 2-day interval.

### Evaluation of EAE symptoms

Mice were weighed and scored daily as follows: 0, no visual sign of disease; 0.5, partial tail paralysis; 1, complete tail paralysis; 1.5, weakness in one hindlimb; 2, weakness in both hindlimbs; 2.5, partial hindlimb paralysis; 3, complete hindlimb paralysis; 3.5, partial forelimb paralysis; 4, complete forelimb paralysis; and 5, dead or killed for humane reasons.

### Cell suspension and flow cytometry

Mice were anesthetized and exsanguinated by cardiac perfusion with saline. Spinal cords were harvested, minced with razor blades in Dulbecco’s phosphate-buffered saline (DPBS, with Ca^2+^ and Mg^2+^), digested for 45 min at 37 °C in DPBS containing 0.13 U/ml Liberase TM (Roche Diagnostics) and 50 U/ml DNase I (Sigma-Aldrich), filtered through 40-μm cell strainers, and then separated from myelin debris by centrifugation in 35 % Percoll (GE Healthcare). The spleens were mashed through 40-μm cell strainers and treated with ammonium chloride solution (Stemcell Technologies) to remove residual erythrocytes. For immunostaining, the cells were incubated sequentially with rat anti-CD16/CD32 antibody (5 μg/ml, BD Biosciences, clone 2.4G2) and Fixable viability dye eFluor 506 (1:1000, eBioscience) for 5 min, with anti-IL-36R antibody (Abcam #ab171844 or R&D Systems #AF2354) for 30 min, and with combinations of the following antibodies for 30 min: rat anti-CD45-FITC (BD Biosciences, clone 30-F11), rat anti-CD11b-V450 (BD Biosciences, clone M1/70), rat anti-Ly6G-APC-Cy7 (Biolegend, clone 1A8), rat anti-CD3ε-PE (BD Biosciences, clone 145-2C11), rat anti-CD19-PerCP-Cy5.5, (BD Biosciences, clone 1D3), rat anti-CD11c-Alexa 647 (Biolegend, clone N418), and goat anti-rabbit IgG-Alexa 594 (Invitrogen, Cat No A11072). The latter antibodies were diluted at 1:200, except anti-CD45-FITC, which was diluted at 1:100. Isotype control antibodies and fluorescence-minus-one controls were used for gating. Cells were washed and resuspended in PBS before being analyzed with a FACSAria II flow cytometer (BD Biosciences). All the analyses were performed by excluding dead cells and doublets using FlowJo software (Tree Star, version 10.0.7r2).

### Western blotting

Ly6G^+^ neutrophils, isolated by flow cytometry, were homogenized in extraction buffer (50 mM Tris-HCl at pH 7.4, 150 mM NaCl, 1 % Triton X-100, 1 mM ethylenediaminetetraacetic acid, 1 mM ethylene glycol tetraacetic acid, 2 mM Na pyrophosphate, 10 mM Na β-glycerophosphate, 1 mM Na orthovanadate, 1 mM phenylmethanesulfonylfluoride, 1× protease and phosphatase inhibitor cocktail [Sigma]). The protein samples (50 μg) were resolved in a 12 % SDS-PAGE Mini-Protean Precast gel (Bio-Rad) and transferred to a polyvinylidene difluoride membrane (PerkinElmer). The membrane was blocked in PBS containing 0.1 % Tween 20 and 7 % non-fat milk, and then incubated at 4 °C overnight with an antibody against IL-36γ (1:200, Santa Cruz, sc-168163) or β-actin (1:50000, Abcam, mab150), followed by 1 h at room temperature in the appropriate secondary horseradish peroxidase-conjugated antibody (Cell Signaling Technology). The antibodies were detected using the Western Lightning Plus-ECL chemiluminescence substrate (Perkin Elmer).

### Immunostaining

Immunostaining was performed as described previously [51] using the following primary antibodies: rat anti-CD3 (1:500, BD Biosciences, clone 17A2) and rat anti-Ly6G (1:5000, BD Biosciences, clone 1A8).

### In situ hybridization

Spinal cord sections were analyzed for IL-36γ and IL-36R mRNAs by radioisotopic in situ hybridization as described previously [52].

### Microscopy

Micrographs were taken using a Retiga EX camera on a Nikon E800 microscope. Images were adjusted for contrast, brightness, and sharpness using Photoshop 12 (Adobe Systems).

### Microglial cell isolation and culture

Primary microglia were isolated from newborn mice (1 day old or less). The brains were minced with razor blades in DPBS and passed four times through a 20-G needle. After centrifugation (1500 rpm), the pellets were resuspended and incubated at 37 °C for 15 min in DPBS containing 0.25 % trypsin-EDTA (Wisent) and 50 U/ml DNase I. The cells were plated in T-25 flasks (~3 brains/flask) and grown in Dulbecco’s modified Eagle’s medium (Wisent) supplemented with 10 % heat-inactivated HyClone bovine growth serum, with medium changed every 2 days. After 2 weeks, the microglia were separated from other glial cells as described previously [53]. Briefly, they were incubated with 0.05 % trypsin in DMEM at 37 °C for 30–45 min, rinsed with PBS to remove contaminating non-adherent cells, then grown in a 1:1 mixture of fresh media and conditioned media from mixed glial cultures (2–3 days). Microglial purity was >99 %, as determined by flow cytometry using the myeloid cell marker CD11b.

For experimentation, primary microglia were seeded in 12-well plates and used at ~70 % confluence (after 5–7 days). BV2 microglia were seeded in 12-well plates at a density of 500,000 cells and used on the next day. Cells were stimulated with 100 ng/ml recombinant mouse IL-36γ (amino acids 13-164, R&D Systems) or PBS as control. After 6 h, the cells were rinsed with PBS, resuspended in lysis buffer and stored at −80 °C until RNA extraction.

### RNA extraction and qRT-PCR

Total RNA from tissues and cells was isolated by homogenization in TRI-reagent or lysis buffer (Sigma-Aldrich), respectively. GenElute Mammalian Total RNA Miniprep Kit (Sigma-Aldrich) was used for RNA purification. First strand cDNA was generated from 5 (tissues) or 1 (cells) μg of total RNA using Superscript III (Invitrogen) with random hexamers and 20-mer oligo-dT primers, then purified using the GenElute PCR Clean-Up Kit (Sigma-Aldrich). The product (20 ng) was analyzed using the LightCycler 480 system with the SYBR Green I Master mix and primers listed in Additional file 2: Table S2 according to the manufacturer’s instructions (Applied Biosystems). The PCR conditions consisted of 45 cycles of denaturation (10 s at 95 °C), annealing (10 s at 60 °C), elongation (14 s at 72 °C), and reading (5 s at 74 °C). The number of mRNA copies was determined using the second derivative method [54].

### Statistical analyses

Data are expressed as mean ± standard error. Means were compared using the Wilcoxon or Kruskal-Wallis test, except for the microarray data, which were compared using Student’s *t* test. EAE incidence curves were constructed using the Kaplan-Meier method and compared using the Wilcoxon test. All these analyses were performed using JMP 10 (SAS Institute) with a significance level of 5 %.

## Results

### Neutrophils express IL-36γ in the CNS and hematopoietic tissues of EAE mice

To compare the spatio-temporal expression patterns of IL-36R and its agonists (IL-36α, IL-36β, IL-36γ) in different MS models, we quantified their mRNAs by quantitative real-time PCR (qRT-PCR) in nervous and hematopoietic tissues from mice killed at various time points after EAE induction by either one of the following methods: (1) active immunization with MOG_35-55_ peptide in complete Freund’s adjuvant (CFA) plus PTX (active EAE), (2) adoptive transfer of splenocytes isolated from EAE mice on day 8 and restimulated in culture for 2 days with MOG_35-55_ in the presence of IL-12 and IL-23 (passive EAE), and (3) intraperitoneal injections of PTX to 2D2 mice expressing a transgenic MOG-specific T cell receptor [55]. Because the latter mice develop EAE within 2 weeks at an incidence lower than 60 % [24, 55], we killed them at a single time point (day 15) and separated them into those that had developed EAE or not (2D2 with or without EAE). Wild-type mice injected with PBS, PTX, or CFA only were used as controls.

**Fig. 1:**
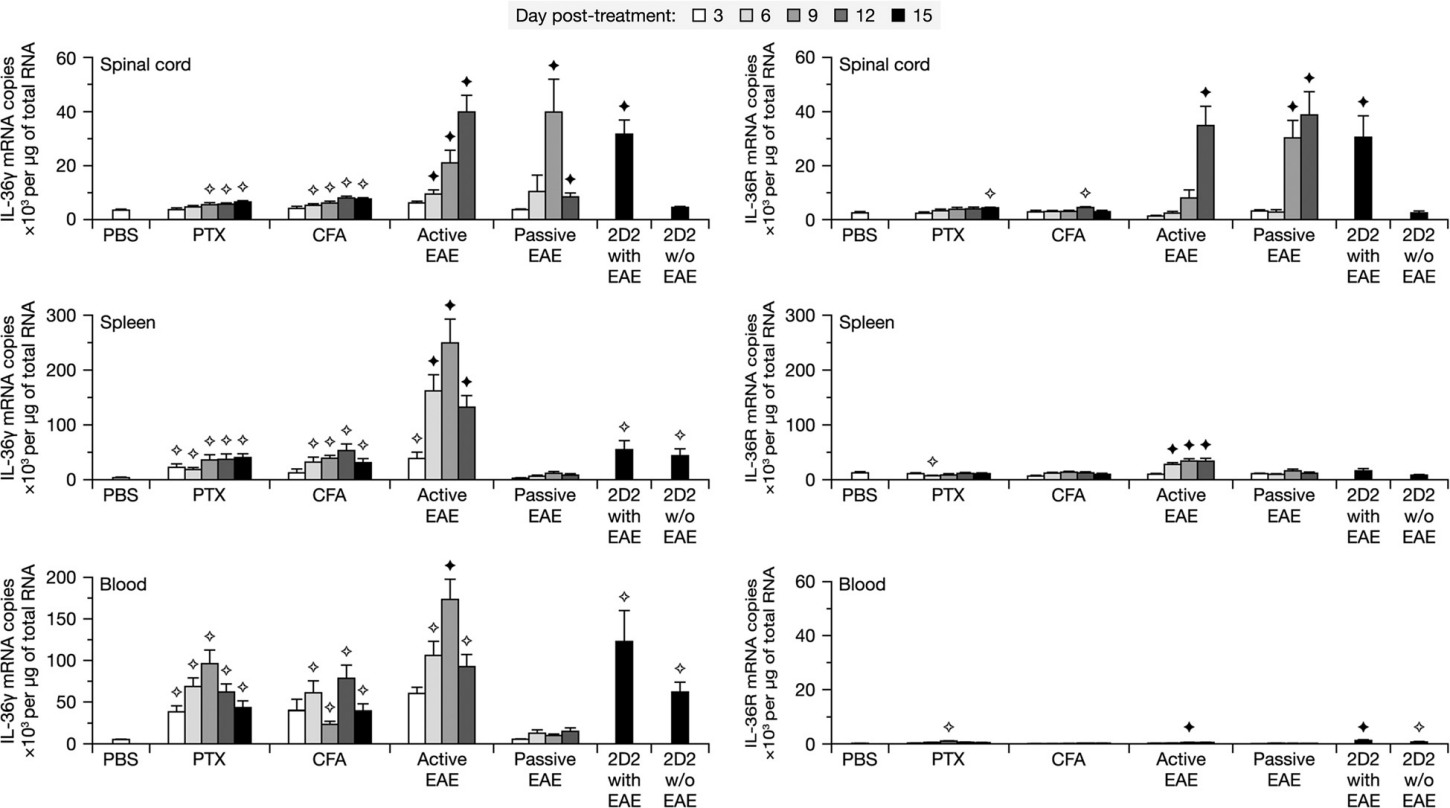
IL-36γ and IL-36R are transcriptionally upregulated in 3 EAE models. Quantification of the mRNAs encoding IL-36γ and IL-36R by qRT-PCR in different tissues from mice killed at the indicated time points after induction of EAE by either active immunization with MOG peptide, passive transfer of encephalitogenic T cells, or injection of PTX to 2D2 mice. Control non-transgenic mice were injected with PBS, PTX, or CFA only. *Stars* indicate significant differences from the PBS group only (*white star*) or all the corresponding control groups (*black star*), as determined by Wilcoxon tests (*P* ≤ 0.038). Sample size: 11–13 (PBS), 5–13 (PTX), 4–8 (CFA), 7–8 (active EAE), 5–8 (passive EAE), 6 (2D2 with EAE), or 5 (2D2 without [*w/o*] EAE)

In the spinal cord, IL-36γ and IL-36R mRNAs were markedly increased in the three EAE models, compared to control mice and PTX-treated 2D2 mice without EAE (Fig. 1). An important difference between the active and passive models is that IL-36γ mRNA peaked at the latest time point examined (day 12) in the former, whereas it peaked at day 9 and declined thereafter in the latter. In the hematopoietic tissues (spleen, blood), IL-36γ mRNA was increased in active EAE and, to a lesser extent, in response to PTX and CFA, but not in passive EAE. The upregulation of IL-36γ was MOG-specific in the spleen and spinal cord, but mainly attributable to the adjuvants in the blood. Higher expression of IL-36γ mRNA was also found in the spleen of 2D2 mice compared to that of wild-type mice, but there was no difference between PTX-treated 2D2 mice with or without EAE. IL-36R mRNA was modestly increased only in the spleen of mice with active EAE. No significant expression of IL-36α and IL-36β mRNAs was detected in any of the tissues and conditions examined (average amounts detected: <220 copies per μm of total RNA; data not shown). Together, these results suggest that IL-36γ is the primary IL-36R agonist that is upregulated during EAE in both CNS and hematopoietic tissues, where its receptor can also be expressed.

**Fig. 2:**
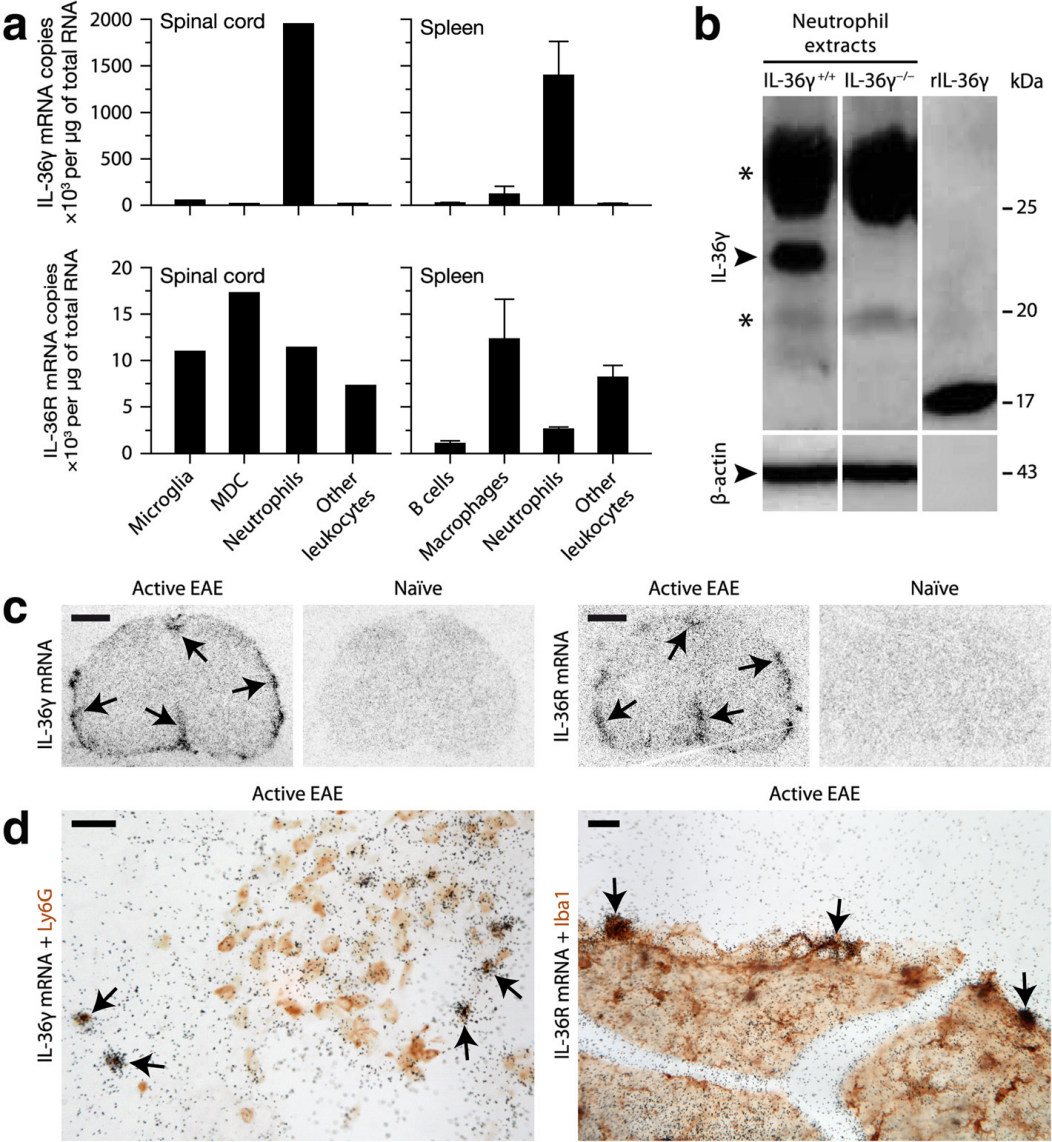
IL-36γ is selectively expressed by neutrophils, while IL-36R is expressed by different leukocytes such as monocytic cells. **a** Quantification of IL-36γ or IL36R mRNA by qRT-PCR in different cells purified from the spinal cord or spleen of EAE mice by FACS using the following gating strategies: neutrophils, Ly6G^+^CD11b^+^CD45^+^CD3^−^CD19^−^; microglia, CD11b^+^CD45^low^Ly6G^−^CD3^−^CD19^−^; monocyte-derived cells (MDC; comprising macrophages and CD11c^+^ dendritic cells), CD11b^+^CD45^+^CD11c^+/−^Ly6G^−^CD3^−^CD19^−^; other intraspinal leukocytes, CD45^+^CD3^+/−^CD19^+/−^CD11b^−^Ly6G^−^CD11c^−^; B cells, CD19^+^CD45^+^CD11b^−^Ly6G^−^CD3^−^; splenic macrophages, CD11b^+^CD45^+^CD11c^−^Ly6G^−^CD3^−^CD19^−^; other splenic leukocytes, CD45^+^CD3^+/−^CD11b^+/−^CD11c^+/−^Ly6G^−^CD19^−^. Sample size: spinal cord, one pooled sample of sorted cells from four mice; spleen, five non-pooled samples from individual mice. **b** Western blotting showing the full-length form of IL-36γ (~22 kDa) in splenic neutrophils from a wild-type EAE mouse (IL-36γ^+/+^), but not from an IL-36γ-deficient EAE mouse (IL-36γ^−/−^). Data are representative of at least four mice per group. The recombinant (*truncated*) form of IL-36γ was used as a control (*right lane*). β-actin (*lower panels*) was used to control for protein loading. *Asterisks* indicate non-specific bands. **c** Autoradiograms showing in situ hybridization signals (*arrows*) for IL-36γ or IL-36R mRNA in the spinal cord of mice killed on day 12 after active EAE induction, but not in naïve mice. Note the submeningeal distribution of the signals (representative of at least five mice). Scale bar = 500 μm. **d** Double labeling for IL-36γ or IL-36R mRNA (*black grains*, in situ hybridization) and cell type-specific markers (*red brown*, immunohistochemistry) in CNS sections from EAE mice. *Arrows* show examples of double-labeled cells. Scale bar = 20 μm

To identify the cell types expressing IL-36γ and IL-36R, we first quantified their mRNAs by qRT-PCR in different leukocytes purified from the spleen or spinal cord of mice with active EAE (day 12) by flow cytometry on the basis of established cell-specific markers. In both tissues, IL-36γ mRNA was detected at high levels only in Ly6G^+^ neutrophils, whereas IL-36R was detected in different cells, especially those of the myeloid lineage (Fig. 2a). Second, the expression of IL-36γ was confirmed at the protein level by Western blotting using FACS-purified splenic neutrophils (note that it was not possible to include neutrophils from spinal cords, because they were less abundant and yielded not enough proteins). A specific band for IL-36γ was detected at the predicted molecular weight of ~22 kDa in neutrophils from IL-36γ^+/+^ mice, but not from IL-36γ^−/−^ mice (Fig. 2b). We also attempted to confirm the expression of IL-36R by flow cytometry using two different antibodies, but none of them provided reliable results, as the labeling was comparable between IL-36R^+/+^ and IL-36R^−/−^ neutrophils and was therefore non-specific (data not shown). Third, we examined the distribution of IL-36γ mRNA in spinal cord sections from EAE and naïve mice by radioisotopic in situ hybridization. Some of these sections were also immunostained for Ly6G or the macrophage/microglia marker Iba1. Both transcripts were strongly expressed in EAE mice in areas near the meninges, but were not detectable in naïve mice (Fig. 2c). IL-36γ mRNA was co-localized with Ly6G^+^ cells, while IL-36R mRNA was reliably co-localized only with Iba1^+^ cells (Fig. 2d), although we could not exclude its presence in other cell types. These results led us to conclude that neutrophils can produce IL-36γ during EAE in both CNS and peripheral compartments, potentially to communicate with microglia and other myeloid cells.

### Microglia respond to IL-36γ by expressing inflammatory genes

**Fig. 3:**
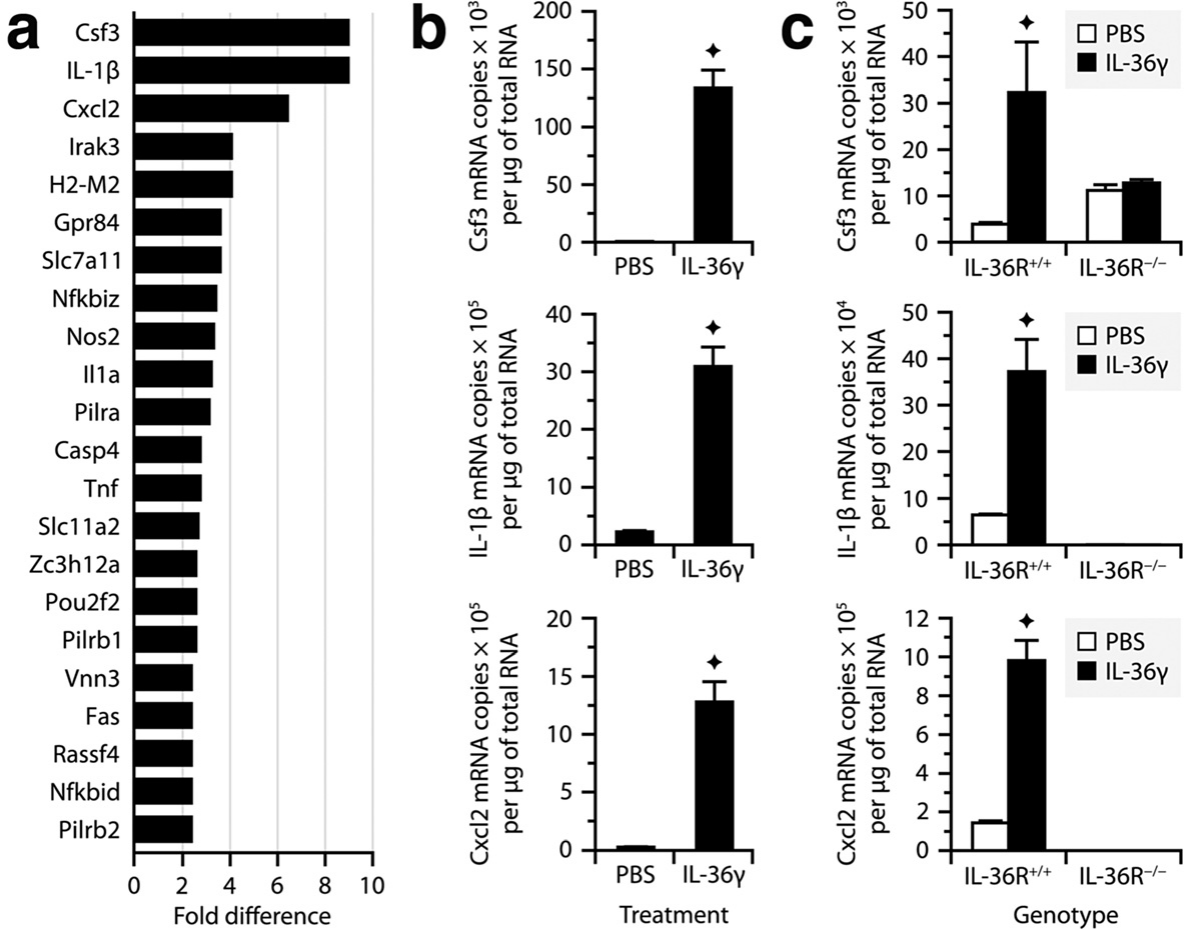
IL-36γ stimulates the expression of inflammatory genes in microglial cultures. **a** Genes that were upregulated ≥2.5 times in BV2 microglia treated for 6 h with recombinant IL-36γ (100 ng/ml) compared to PBS, as determined using Affymetrix DNA microarrays (Student’s *t* test, *P* < 0.04). **b**, **c** Quantitative PCR analysis confirming that Csf3, IL-1β, and Cxcl2 mRNAs were upregulated by IL-36γ in BV2 cells (**b**) and primary microglia from IL-36R^+/+^ mice (**c**) (Wilcoxon test, *P* < 0.005). Note in **c** the absence of upregulation in primary microglia from IL-36R^−/−^ mice, confirming the specificity of the results

To demonstrate that microglia are adequately equipped to respond to IL-36γ, we first compared the transcriptional profiles of BV2 microglial cells treated or not for 6 h with recombinant IL-36γ using Affymetrix DNA microarrays. The dose used (100 ng/ml) was chosen because it was previously reported to be optimal for stimulating IL-6 secretion from cultured dendritic cells [38]. Twenty-two genes were found to be upregulated ≥2 times with a hybridization signal ≥100 (Additional file 3: Table S3 and Fig. 3a). The three most upregulated genes coded for Csf3, IL-1β, and Cxcl2, which are well known to be involved in neutrophil biology [56]. Quantitative PCR analysis confirmed that these 3 genes were strongly upregulated in BV2 cells by IL-36γ (Fig. 3b). To validate these results, we repeated the experiment with primary microglia collected from IL-36R^+/+^ and IL-36R^−/−^ mice. As expected, upregulation of Csf3, IL-1β, and Cxcl2 were observed in IL-36R^+/+^ microglia following IL-36γ exposure, but not in IL-36R^−/−^ microglia (Fig. 3c). The latter cells were not devoid of the ability to express these genes because they expressed them at the same levels as IL-36R^+/+^ microglia when stimulated with lipopolysaccharide (10 ng/ml; data not shown). Altogether, these results indicate that microglia have the potential to respond to IL-36γ, at least ex vivo.

### The IL-36γ–IL-36R axis does not influence the course of active EAE

**Fig. 4:**
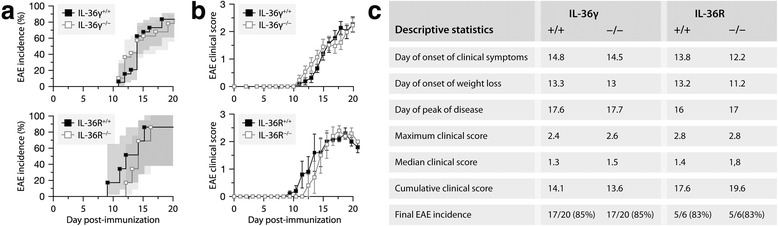
Neither IL-36γ nor IL-36R is required for EAE development. **a** Kaplan-Meier curves showing EAE incidence in mice expressing (*black squares*) or lacking (*white squares*) IL-36γ (*upper graph*) or IL-36R (*bottom graph*) after immunization with MOG. The graphs include all the mice tested. No significant intergenotype difference was detected (Wilcoxon tests, *P* ≥ 0.41). Sample size: 20 (IL-36γ^+/+^), 20 (IL-36γ^−/−^), 6 (IL-36R^+/+^), or 6 (IL-36R^−/−^). **b** EAE severity in mice expressing (*black squares*) or lacking (*white squares*) IL-36γ (*upper graph*) or IL-36R (*bottom graph*). The graphs include only mice that had developed clinical signs of EAE at the end of the study (i.e., 21 days). No significant intergenotype difference was detected (Wilcoxon tests, *P* ≥ 0.12). Sample size: 17 (IL-36γ^+/+^), 17 (IL-36γ^−/−^), 5 (IL-36R^+/+^), or 5 (IL-36R^−/−^). **c** Additional statistics for EAE in mice expressing (+/+) or not (−/−)IL-36γ or IL-36R. No significant intergenotype difference was detected in any of these parameters (Wilcoxon tests, *P* ≥ 0.34). Sample size: 20 (IL-36γ^+/+^), 20 (IL-36γ^−/−^), 6 (IL-36R^+/+^), or 6 (IL-36R^−/−^)

**Fig. 5:**
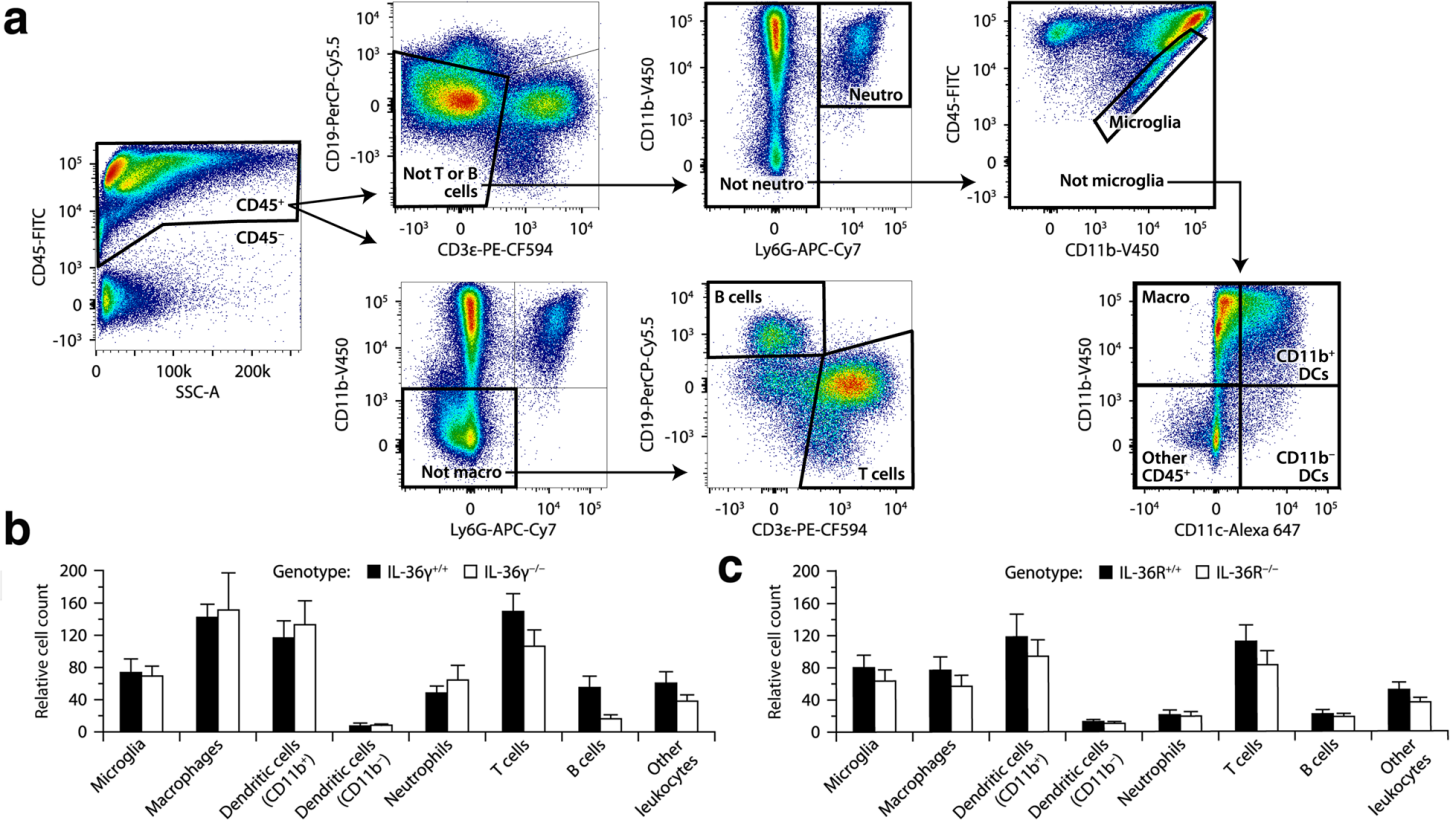
No difference in leukocyte recruitment in the spinal cord of EAE mice expressing or not IL-36γ or IL-36R. **a** Gating strategy used for flow cytometric analysis. Dead cells and doublets were excluded. The data shown are from a representative wild-type EAE mouse. **b**, **c** Counts of immune cells in the spinal cord of IL-36γ^−/−^, IL-36R^−/−^, or wild-type mice killed 21 days after immunization with MOG peptide. The counts were normalized to CD45^−^ cells used as an internal control. When the cells were collected, the animals had clinical scores ranging from 0.5 to 3. No significant difference was found between the genotypes (Wilcoxon tests, *P* ≥ 0.07). Sample size: 6 (**b**) or 9 (**c**) mice per group

As IL-36γ and IL-36R are strongly and chronically expressed in active EAE (Figs. 1 and 2) and as the resident macrophages of the CNS can potentially respond to IL-36γ (Fig. 3), we hypothesized that the loss of these proteins would have an impact on the clinical course of active EAE. To address this possibility, we induced the disease in mice deficient or not in either IL-36γ or IL-36R and then scored their symptoms daily for 21 days. As shown in Fig. 4, the incidence, onset, and severity of EAE were similar between the knockouts and their wild-type controls. To examine whether the genetic deletions had influenced the nature of the immune cells infiltrating the CNS, we killed all of the mice on day 21 and prepared single-cell suspensions from their spinal cords for flow cytometric analysis. Consistently with the behavioral observations, no intergenotype difference was observed in the number of microglia, macrophages, dendritic cells, neutrophils as well as of T and B cells (Fig. 5). Therefore, these results demonstrate that the IL-36γ-IL-36R axis does not play a significant role in the autoimmune response involved in active EAE.

## Discussion

As observed in other immunological diseases such as psoriasis [29, 32, 34, 57, 58], the present study shows that IL-36γ and IL-36R are upregulated in the three models of EAE. It also shows that IL-36γ derives from neutrophils and can stimulate microglia to produce neutrophil-stimulating cytokines, which is consistent with previous studies showing that IL-36γ induces both the production of such cytokines in other myeloid cells residing outside the CNS [31, 38, 40–42] and the recruitment of neutrophils [42]. Despite these observations, we demonstrate that neither IL-36γ nor IL-36R plays a significant role in EAE, similarly to what was reported in murine models of arthritis [59, 60] and mycobacterial infection [61].

The main question that arises is why IL-36γ is expressed, but does not play a role in certain conditions. One hypothesis is that IL-36γ, like other members of the IL-1 family, requires a second signal to be processed, activated, and secreted. This signal would be present in psoriasis, but not in EAE and arthritis models. In support of this, it has been shown that IL-36γ requires N-terminal truncation to be fully active [27]. However, it is unknown whether this truncation occurs in vivo and what would be the protease involved. Our work shows, for the first time to our knowledge, that the full-length IL-36γ protein (193 amino acids, 22 kDa) is detectable in vivo (i.e., in neutrophils from EAE mice). As the truncated form is not detectable in the same conditions, it is likely that IL-36γ remains stored under a pro-form in the cytoplasm of neutrophils during EAE. A second (non-exclusive) hypothesis is that neutrophils release IL-36γ during a cell death process such as NETosis, which occurs in psoriasis [62], but not likely in EAE. This possibility that IL-36γ acts as an alarmin (i.e., an endogenous molecule that signals tissue and cell damage) is so far only supported by the observation that the TLR3 agonist polyinosinic-polycytidylic acid concomitantly induces cell death and IL-36γ release in keratinocyte cultures [35]. Finally, a third hypothesis is that the CNS, an immunoprivileged site, has a higher activation threshold to IL-36γ due to a combination of mechanisms (e.g., via the secretion of IL-36 antagonists and the generation of intracellular signaling inhibitors such as SIGIRR [63]).

A positive aspect of this study is that we have identified microglia as a potential target of IL-36γ. This suggests that IL-36γ could contribute to neuroinflammation, perhaps by promoting neutrophil recruitment, in certain conditions that remain to be determined. Another positive aspect is that we have identified neutrophils as a major and exclusive source of IL-36γ in nervous and hematopoietic tissues (i.e., brain, spinal cord, spleen, blood). According to previous studies, keratinocytes and other epithelial cells can also produce IL-36γ in culture [30–34, 64], but, to our knowledge, this remains to be confirmed in vivo. Therefore, it will be important in the future to determine whether neutrophils are a predominant source of IL-36γ in epithelial inflammatory diseases (e.g., psoriasis, asthma) as they are in EAE.

In conclusion, this study demonstrates that components of the IL-36 signaling pathway are strongly expressed in EAE, but that they do not contribute to this pathology. Furthermore, by revealing that IL-36γ is a neutrophil-specific marker in different tissues and that microglia have the potential to response to this cytokine, this study opens up new research avenues for elucidating the biological function of IL-36γ and its clinical significance.

## Additional files

Additional file 1: Table S1. Primers used for genotyping. (PDF 39 kb) Additional file 2: Table S2. Primers used for qRT-PCR. (PDF 38 kb) Additional file 3: Table S3. Genes that were upregulated ≥2 times in BV2 microglial cells cultured for 6 h with recombinant IL-36γ (100 ng/ml), as determined using Affymetrix DNA microarrays. (PDF 70 kb)
