# Supplementary material for: Interleukin-36γ is expressed by neutrophils and can activate microglia, but has no role in experimental autoimmune encephalomyelitis
Source: J Neuroinflammation. 2015 Sep 17;12:173. doi: 10.1186/s12974-015-0392-7 (PMC4574267; doi:10.1186/s12974-015-0392-7)
Supplement: Additional file 1: Table S1. — Primers used for genotyping. (PDF 39 kb) [file 12974_2015_392_MOESM1_ESM.pdf]

Supplementary Table 1. Primers used for genotyping.

Mouse strain	Allele	Forward primer	Reverse primer
2D2	Transgene	5'-cccgggcaaggctcagccatgctcctg-3'	5'-gcggccgcaattcccagagacatccctcc-3'
	Internal control	5'-ctaggccacagaattgaaagatct-3'	5'-gtaggtggaaattctagcatcatcc-3'
IL-36γ$^{-/-}$	Mutated	5'-ggcggatttctgagttggag-3'	5'-gcagcgcatcgccttctatc-3'
	Wild-type	5'-ctgggctatttgtatcttca-3'	5'-cacacctgctggtccaagtc-3'
IL-36R$^{-/-}$	Mutated	5'-gccctgaatgaactgcaggacg-3'	5'-cacgggtagccaacgctatgtc-3'
	Wild-type	5'-gccgctacacaccacaaccag-3'	5'-agttcagtagtccactgccactc-3'
