# Supplementary material for: Interleukin-36γ is expressed by neutrophils and can activate microglia, but has no role in experimental autoimmune encephalomyelitis
Source: J Neuroinflammation. 2015 Sep 17;12:173. doi: 10.1186/s12974-015-0392-7 (PMC4574267; doi:10.1186/s12974-015-0392-7)
Supplement: Additional file 2: Table S2. — Primers used for qRT-PCR. (PDF 38 kb) [file 12974_2015_392_MOESM2_ESM.pdf]

Supplementary Table 2. Primers used for qRT-PCR.

mRNA	Forward primer	Reverse primer
Csf3	5'-aagctgtgtcaccccgaggag-3'	5'-tggagctggcttaggcactgt-3'
Cxcl2	5'-atgcctgaagaccctgccaag-3'	5'-ggtcagttagccttgcctttg-3'
IL-1β	5'-tcaaatctcgcagcagcacatc-3'	5'-ccagcaggttatcatcatcatccc-3'
IL-36R	5'-aaggaagttgagtatggaagaaggatc-3'	5'-ggtttgtattctccttcgtgtctg-3'
IL-36α	5'-ttccagtcactattaccttgctccc-3'	5'-gctccccatcctttgtgcaga-3'
IL-36β	5'-ggctttccctccacaatcttg-3'	5'-acccataccatctgttgtgagtca-3'
IL-36γ	5'-caggcccttgtgacagttcca-3'	5'-ttagcagcaaagtagggtgtccatta-3'
