# Supplementary material for: Interleukin-36γ is expressed by neutrophils and can activate microglia, but has no role in experimental autoimmune encephalomyelitis
Source: J Neuroinflammation. 2015 Sep 17;12:173. doi: 10.1186/s12974-015-0392-7 (PMC4574267; doi:10.1186/s12974-015-0392-7)
Supplement: Additional file 3: Table S3. — Genes that were upregulated ≥2 times in BV2 microglial cells cultured for 6 h with recombinant IL-36γ (100 ng/ml), as determined using Affymetrix DNA microarrays. (PDF 70 kb) [file 12974_2015_392_MOESM3_ESM.pdf]

Supplementary Table 3. Genes that were up-regulated ≥ 2 times in BV2 microglial cells cultured for 6 h with recombinant IL-36γ (100 ng/ml), as determined using Affymetrix DNA microarrays.

			Hybridization signal				Mean		Fold difference	
**Probe Set ID**	**Gene Symbol**	**Gene Description**	**PBS**	**PBS**	**IL-36γ**	**IL-36γ**	**PBS**	**IL-36γ**	**IL-36γ/PBS**	**T test (*P*)**
17256129	Csf3	Colony stimulating factor 3 (granulocyte)	60,9	53,3	541,0	501,4	57,1	521,2	9,1	0,0019
17391565	IL-1β	Interleukin 1 beta	759,6	705,5	6437,9	6834,3	732,6	6636,1	9,1	0,0011
17438995	Cxcl2	Chemokine (c-x-c motif) ligand 2	73,2	53,3	377,5	442,5	63,3	410,0	6,5	0,0095
17245399	Irak3	Interleukin-1 receptor-associated kinase 3	212,5	206,8	845,3	889,8	209,7	867,5	4,1	0,0012
17344873	H2-M2	Histocompatibility 2, m region locus 2	251,9	249,9	1034,2	1014,5	250,9	1024,3	4,1	0,0002
17322355	Gpr84	G protein-coupled receptor 84	378,0	429,5	1438,4	1655,1	403,8	1546,7	3,8	0,0094
17405082	Slc7a11	Solute carrier family 7 (cationic amino acid transporter,	204,9	198,2	780,8	741,0	201,6	760,9	3,8	0,0013
17330967	Nfkbiz	Nuclear factor of kappa light polypeptide gene enhanc	162,2	195,1	684,3	615,4	178,7	649,8	3,6	0,0065
17253707	Nos2	Nitric oxide synthase 2, inducible	1060,9	965,7	3626,0	3497,8	1013,3	3561,9	3,5	0,0010
17391554	Il1a	Interleukin 1 alpha	2531,2	2520,7	8375,6	8810,6	2526,0	8593,1	3,4	0,0013
17454166	Pilra	Paired immunoglobin-like type 2 receptor alpha	580,3	599,8	1874,4	2018,9	590,1	1946,6	3,3	0,0029
17514435	Casp4	Caspase 4, apoptosis-related cysteine peptidase	386,8	422,0	1168,2	1183,8	404,4	1176,0	2,9	0,0006
17344309	Tnf	Tumor necrosis factor	1003,0	1004,4	2779,0	3031,8	1003,7	2905,4	2,9	0,0044
17321683	Slc11a2	Solute carrier family 11 (proton-coupled divalent metal	442,1	419,7	1158,0	1216,4	430,9	1187,2	2,8	0,0017
17429933	Zc3h12a	Zinc finger ccch type containing 12a	222,1	261,7	686,3	637,4	241,9	661,9	2,7	0,0056
17487855	Pou2f2	Pou domain, class 2, transcription factor 2	122,5	130,2	387,2	300,3	126,4	343,7	2,7	0,0380
17454179	Pilrb1	Paired immunoglobin-like type 2 receptor beta 1	339,2	396,3	1027,4	927,9	367,8	977,7	2,7	0,0087
17232152	Vnn3	Vanin 3	98,7	98,7	268,0	232,1	98,7	250,0	2,5	0,0138
17358797	Fas	Fas (tnf receptor superfamily member 6)	52,3	52,7	148,6	115,6	52,5	132,1	2,5	0,0404
17470060	Rassf4	Ras association (ralgds/af-6) domain family member 4	446,3	434,9	1108,0	1073,5	440,6	1090,8	2,5	0,0008
17476372	Nfkbid	Nuclear factor of kappa light polypeptide gene enhanc	443,9	464,9	1111,3	1128,5	454,4	1119,9	2,5	0,0004
17454187	Pilrb2	Paired immunoglobin-like type 2 receptor beta 2	72,9	56,3	150,2	166,8	64,6	158,5	2,5	0,0153
17432674	Tnfrsf1b	Tumor necrosis factor receptor superfamily, member 1	1799,9	1801,9	4489,3	4215,5	1800,9	4352,4	2,4	0,0029
17481556	Ppfibp2	Ptprf interacting protein, binding protein 2 (liprin beta 2	75,8	86,6	210,9	180,7	81,2	195,8	2,4	0,0190
17302475	Irg1	Immunoresponsive gene 1	3196,1	2986,0	7496,1	7238,8	3091,1	7367,5	2,4	0,0015
17218060	Ptgs2	Prostaglandin-endoperoxide synthase 2	1862,7	2013,7	4551,6	4483,1	1938,2	4517,4	2,3	0,0010
17406279	Tlr2	Toll-like receptor 2	202,3	198,5	465,2	465,8	200,4	465,5	2,3	0,0001
17470627	Clec4e	C-type lectin domain family 4, member e	1068,3	1082,4	2436,3	2519,0	1075,4	2477,6	2,3	0,0009
17508523	Rab11fip1	Rab11 family interacting protein 1 (class i)	183,0	181,7	420,1	418,3	182,4	419,2	2,3	0,0000
17459196	Tnip3	Tnfaip3 interacting protein 3	1451,5	1317,5	3090,0	3274,1	1384,5	3182,1	2,3	0,0040

			Hybridization signal				Mean		Fold difference	
Probe Set ID	Gene Symbol	Gene Description	PBS	PBS	IL-36γ	IL-36γ	PBS	IL-36γ	IL-36γ/PBS	T test (*P*)
17281219	Nfkbia	Nuclear factor of kappa light polypeptide gene enhanc	639,1	618,4	1485,1	1396,0	628,8	1440,6	2,3	0,0032
17254047	Ccl7	Chemokine (c-c motif) ligand 7	1613,9	1672,5	3742,5	3739,8	1643,2	3741,2	2,3	0,0002
17266157	Cpd	Carboxypeptidase d	890,1	956,8	2048,9	2087,2	923,5	2068,1	2,2	0,0011
17512809	Hp	Haptoglobin	278,3	280,8	604,0	628,6	279,6	616,3	2,2	0,0013
17254166	Slfn2	Schlafen 2	496,6	534,3	1114,8	1110,9	515,5	1112,9	2,2	0,0010
17309981	Fyb	Fyn binding protein	540,2	532,6	1128,9	1172,2	536,4	1150,5	2,1	0,0013
17548123	Ehd1 // Ehd1	Eh-domain containing 1 // eh-domain containing 1	63,3	61,8	129,6	124,6	62,6	127,1	2,0	0,0016
17226771	Ikbke	Inhibitor of kappab kinase epsilon	453,0	495,5	983,3	934,8	474,3	959,1	2,0	0,0044
17262855	Tnip1	Tnfaip3 interacting protein 1	651,0	662,6	1336,4	1317,5	656,8	1326,9	2,0	0,0003
17410542	Nfkb1	Nuclear factor of kappa light polypeptide gene enhanc	683,1	717,3	1425,9	1399,4	700,2	1412,7	2,0	0,0009
